# Wound Healing Effects of *Dracontomelon dao* on Bacterial Infection Wounds in Rats and Its Potential Mechanisms under Simulated Space Environment

**DOI:** 10.1155/2022/4593201

**Published:** 2022-06-24

**Authors:** Jianxia Wen, Zhuo Xu, Xiao Ma, Yanling Zhao

**Affiliations:** ^1^School of Food and Bioengineering, Xihua University, Chengdu, China; ^2^Department of Pharmacy, Chinese PLA General Hospital, Beijing, China; ^3^School of Pharmacy, Chengdu University of Traditional Chinese Medicine, Chengdu, China

## Abstract

*Dracontomelon dao* (*D*. *dao*) is the leaves of *Dracontomelon duperreanum* Pierre (*D*. *dao* auct. non (Blanco) Merr. and Rolfe; *D. sinense* Stopf.). As a valuable traditional Chinese medicine from Anacardiaceae, *D. dao* has a long history of treating bedsores, skin ulcers, and other infection diseases. In addition, the volatile oil from *D. dao* leaves exhibits antitumor effects. However, these reported studies only focused on evaluating the antimicrobial efficacy on model strains *in vitro*, without paying attention to the antimicrobial activity and anti-inflammatory effects *in vivo*. This study was aimed to provide evidence of antimicrobial activity and anti-inflammatory and proangiogenesis activities of *Dracontomelon dao* (*D. dao*) on the skin of rats under simulated space environment. The weightlessness model of rats in space environment was established. Then, rats were given *D. dao* for 15 days. Wound healing effects of *D. dao* on histopathology and inflammatory cytokines in *E. coli*-induced wound infection in weightless rats were analyzed. Furthermore, the molecular biology technology was performed to evaluate the wound healing effects of *D. dao* on the relative protein level of NF-*κ*B as well as PI3K/Akt signaling pathways. Immunohistochemistry was used for the protein expression of VEGFA. The wound healing effects of *D. dao* on bacterially infected wounds in rats were manifested by lowering the size of the wound and significantly increasing the shrinkage rate of the wound. *D. dao* had effect on alleviating histological damage of skin tissue and downregulation inflammatory cytokines level. In addition, the results indicated that *D. dao* has a regulatory effect on inflammation and angiogenesis and could regulate the relative protein level of MAPK/NF-*κ*B as well as PI3K/AKT signaling pathways. The current study highlighted the crucial role of *D. dao* in relieving skin tissue injury in *E. coli*-induced wound infection in weightless rats by regulating the MAPK/NF-*κ*B as well as PI3K/AKT signaling pathways. This study could provide a new agent for the treatment of bacterial infected wounds in simulated space environment.

## 1. Introduction

People have developed a strong interest in manned spaceflight due to the efforts of private and government agencies [1]. Living in such an extreme environment will result in reduced immune status and profound changes in the human bacterial community. Under microgravity conditions, the efficacy of antibiotics decreases and the mutation rate of microorganisms increases significantly [2, 3]. Assuming that there is no obvious microbial contamination in the structure of the spacecraft or its air, food, and water supply, any infection of the crew members will be caused by endogenous human and animal flora carried at the time of departure, which may limit the types of infection encountered in a predictable manner [4, 5]. It is reported that the microflora of bacteria was found after a short flight, which may be related to diet, mainly intestinal microflora, such as *Escherichia coli* (*E. coli*) [6, 7]. Microgravity environment makes its mutation more resistant [8]. The negative effects of space travel on immune function, especially cellular immunity, will result in the increase of the chances of bacteria building infection lesions [9]. These factors will affect effective treatment and infection diseases will occur. Trauma, such as tearing and open fractures, is likely to occur during the long-term mission in the space station [10]. Once infection wounds occur, it will pose a direct threat to the lives of astronauts and even result in the failure of space missions. Therefore, effective antibiotics are needed to prevent serious wound infections. Many studies have indicated that plant products are potential wound healing and effective antimicrobial agents and are largely more popular because of their wide availability and unnecessary side effects [11–13].

*Dracontomelon dao* (*D. dao*) is the leaves of *Dracontomelon duperreanum* Pierre (*D. dao auct. non* (Blanco) Merr. And Rolfe; *D. sinense* Stopf.). As a valuable traditional Chinese medicine from Anacardiaceae, *D. dao* has a long history of treating bedsores, skin ulcers, and other infection diseases [14, 15]. In addition, the volatile oil from *D. dao* leaves exhibits antitumor effects [16]. Our previous studies [17–19] have shown that different extracts of *D. dao* leaves show different antimicrobial activities, especially the ethyl acetate (EtOAc) extract containing flavonoids and phenolic acids, and exhibit effective antimicrobial activity against *Escherichia coli* (*E. coli*), *Pseudomonas aeruginosa* (*P. aeruginosa*), and *Staphylococcus aureus* (*S. aureus*). However, these reported studies only focused on evaluating the antimicrobial efficacy on model strains *in vitro*, without paying attention to the antimicrobial activity and anti-inflammatory effects *in vivo*. In order to fully imitate the status and living conditions of astronauts in the space capsule, tail-suspended hindlimb-unloaded rats in simulation capsules have been widely performed on for simulating the effects of microgravity. Moreover, this model could lead to body fluids moving to the neck as well head area and induces postural muscle unloading under microgravity condition.

In this study, we investigated the healing of infection wounds in tail-suspended hindlimb-unloaded rats by topical application of *D. dao* under simulated weightlessness environment of space and tried to explore its molecular mechanism. The results indicated that topical application of *D. dao* could not only improve the healing of infection wounds by reducing the expression level of proinflammatory factors, including interleukin-6 (IL-6), interleukin-1*β* (IL-1*β*), and tumor necrosis factor-*α* (TNF-*α*), but also increase angiogenesis by increasing the expression level of growth factors, such as vascular endothelial growth factor A (VEGFA) and transforming growth factor-*β* (TGF-*β*1), thereby shortening healing time. In order to determine the target pathway of *D. dao*, the effects on signaling pathways of nuclear factor kappa-B (NF-*κ*B), phosphoinositide 3-kinase (PI3K), and mitogen-activated protein kinase (MAPK) were subsequently studied to elucidate its related molecular mechanisms.

## 2. Materials and Methods

### 2.1. Ethic Statement

This study was performed in line with the recommendation of the Guidelines for the Care and Use of Laboratory Animals of the Ministry of Science and Technology of China. All breeding and experiments were undertaken with review and approval from the Animal Ethical and Experimental Committee of the Chinese PLA General Hospital. The Approval ID is IACUC-2020-0027.

### 2.2. Preparation of the *D. dao* Extracts

The leaves of *D. dao* (batch number: 20141013) were obtained from the Chinese herbal medicine market in Guangdong Province, China. The leaves were dried in a cool place and stored at room temperature. *D. dao* leaves were roughly grinded in a chalking machine, sifted (60 mesh), and stored in the sealed containers. 540 g of *D. dao* leaves powder was accurately weighed and refluxed with 12 times 80% ethanol for 2 h, which was repeated three times. Then, the extracts were combined, filtered, and evaporated. In addition, they could be dissolved in proper amount of ultrapure water. Finally, the EtOAc extract was obtained by repeated extraction with EtOAc in the ratio of 1 : 1.5 (H_2_O : EtOAc = 1 : 1.5) for 5 times. Finally, the weight ratio of *D. dao* leaves was 12.65%. The extract was dried at low temperature and stored at 4°C for further study.

### 2.3. Animals Handling

A total of 84 healthy inbred Sprague Dawley rats (190–210 g) of both sexes were obtained from SPF Biotechnology Co., Ltd., Beijing, China. They were individually housed and were periodically weighed once a week. All the animals were closely observed for any infection. Rats showing signs of infection would be isolated and excluded from the study. They were maintained in a temperature-controlled room (23 ± 2°C) and kept on a 12 h/12 h light/dark illumination cycle (lights on at 06 : 00 am) and humidity of 45–50% in an air-conditioned room. Rats had free access to food and water. Rats were handled after adapting to the laboratory environment and observation for 7 days.

### 2.4. Weightlessness Model in the Simulated Space Environment

**Figure 1:**
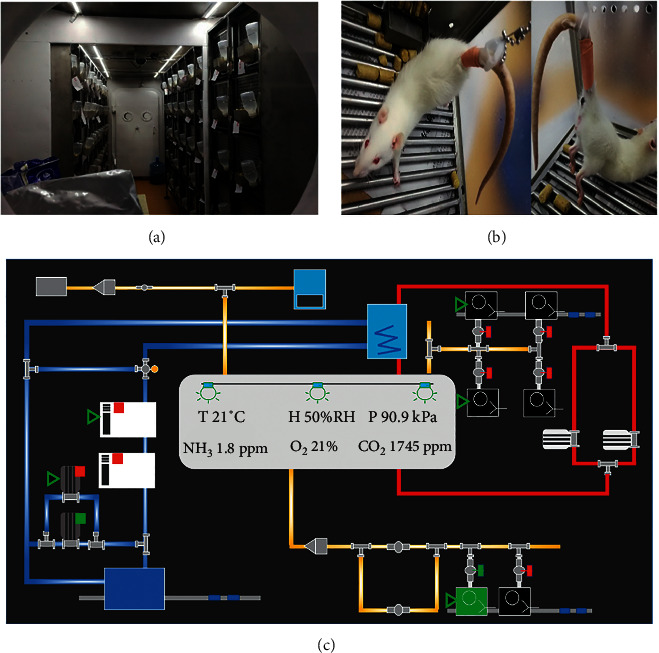
Simulated animal experiment scene in space environment and spacecraft drug screening platform. (a) The scene of animal experiments under the simulated space environment. (b) Hindlimb-suspended rats. (c) Simulated spacecraft drug screening platform.

The tails of the rats were connected to a rotating suspension device mounted on top of a custom-designed plexiglass cage (length = 45 cm, width = 45 cm, height = 45 cm). After washing with 75% ethanol, the tails were fixed with a tail strap that raised the hindlimbs of rats off the cage floor by a 30° head-down angle with their hindlimbs being unloaded [20–22]. They were caged separately and fed on tap water and chow ad libitum (Figures 1(a) and 1(b)). The cages containing rats were placed on a platform outfitted with an oscillator to simulate the capsule's ascent. The vibration conditions were listed as follows: frequency of 91.5 HZ, vibration time of 58 s, and amplitude of 1 G. Subsequently, all animals were fed with a standard laboratory food and raised in the simulated capsule platform of the China Astronaut Research and Training Centre (Beijing, China). The conditions of the platform are shown in Figure 1(c). The simulated space station environment was kept at 18–25°C and 90 ± 1 Kpa on a 45 : 90 min light/dark cycle. The weightless rats were observed for 14 days before the creation of wound. The system is stable and reliable when creating a model of weightlessness in a simulated space environment in this study.

### 2.5. Creation of Wound Infection in Weightless Rats

The weightless rats were depilated on the back after anesthesia with urethane (30%, 0.7 ml/100 g). Two excised wounds were created by cutting a full-thickness skin with a diameter of 1.5 cm from a predetermined area on both sides of the dorsal region of the midline to the depth of loose subcutaneous tissue [23]. The *E. coli* suspension (ATCC 25922, 10^8^ cfu/ml) was dropped via pipette to induce skin infection [24, 25]. 1 ml of the suspension was used for each wound except the uninfected group. The wound was covered with medical transparent film, wrapped, and fixed with sterile antilicking gauze.

### 2.6. Grouping and Administration

The rats were randomly assigned to four groups, each of which contained 21 animals. The dose of the drug was consistent with the results of the previous experimental study [26]:

1. Infection group (IG): the infection wound administered vehicle (30% glycerine) only
2. Low-dosage group (LDG): the infection wound treated with 0.08 g/ml *D. dao* (with 30% glycerine as vehicle)
3. High-dosage group (HDG): the infection wound treated with 0.8 g/ml *D. dao* (with 30% glycerine as vehicle)
4. Uninfection group (UIG): uninfected wound administered vehicle only

After the successful establishment of the skin infection model, the rats were given normal saline for debridement treatment. Each wound was smeared to administer 100 *μ*L corresponding drug liquid once a day for 15 days. Rats had free access to food and water throughout the whole experiment. The doses of *D. dao* used in this research were proved to have no toxic reactions in rats. Subsequently, the wounds were left undressed and placed in an open environment.

### 2.7. Wound Contraction Evaluation

At 0, 3, 5, 10, and 15 days after inflicting the wounds, the wound area was metered by tracing the outline with transparent paper. If the moist granulation tissue was no longer visible and the wound was covered by new epithelial tissue, the wound was supposedly closed (completely healed). The area within each tracing boundary was scanned by a scanner and analyzed by the image analysis software Image-Pro Plus 6.0 (Media Cybernetics, Inc., Bethesda, MD, USA) and expressed as the percentage of wound shrinkage. These values were expressed as a percentage of the 0-day measurement value and were evaluated by Wilson's formula [27]. The formula was as follows:

(1) $$\begin{array}{c} \%\text{wound contraction}=\frac{\text{wound area on }0\text{ day}–\text{wound area on particular day}}{\text{wound area on }0\text{ day}}\times 100\text{\%}. \end{array}$$

### 2.8. Tissue and Blood Collection

Seven rats in each subgroup were selected randomly to collect tissue and blood samples on days 5, 10, and 15. After anesthesia, the rats were punctured at the abdominal aorta to collect blood samples, which were centrifuged to obtain serum. Then, the serum was dispensed into tubules and stored in a refrigerator at −80°C. Next, the granulation tissue and/or healing tissue was collected. One portion of the tissue was preserved in 10% neutral buffered formalin for tissue histopathological and immunohistochemical analysis. The other section was stored at −80°C for further use.

### 2.9. Histopathologic Evaluation

Hematoxylin-eosin staining (HE) was used to detect the histopathological changes of excised wounds in rats. Different concentrations of ethanol were used to hydrate the wound tissue. The excised wound tissue was fixed and sectioned, and the xylene solution was deparaffinized and transparent. Subsequently, the embedded granulation tissue is sliced into thin slices, which were five-micron-thick slices of the epidermis, dermis, and subcutaneous pannus on the glass slide. After dewaxing, the sample was rehydrated with distilled water and stained with hematoxylin and eosin. The sample was placed under a microscope for microscopic examination, and images were collected and analyzed.

### 2.10. Detection of Inflammatory Cytokines

The serum samples of rats were taken out and rethawed in a refrigerator at 4°C. Serum biochemical indices were measured respectively by the Synergy H1 Hybrid Reader (Biotech, USA). The corresponding enzyme-linked immunosorbent assay (ELISA) kits were used to detect the content of serum inflammation cytokines, including TNF-*α*, IL-1*β*, and IL-6. The ELISA kits were purchased from Shanghai MLBIO biotechnology Co., Ltd. (Shanghai, China). The operation process was carried out in strict accordance with the requirements of the ELISA kit instructions. The results were averaged and expressed as pg/mL protein.

### 2.11. Western Blotting for the Protein Expression

The western blotting method was performed to investigate the effect of *D. dao* on the relative protein expression of NF-*κ*B, MAPK, and PI3K/Akt signaling pathways and growth factors. The granulation tissues of rats were homogenized and subsequently analyzed by tissue lyser (Shanghai Jingxin Industrial Development Co., Ltd., Shanghai, China) supplemented with radio immunoprecipitation assay (RIPA) buffer containing phenylmethylsulfonyl fluoride (PMSF). Protein concentration was calculated by the bicinchoninic acid assay (BCA) protein assay kit (Beyotime Biotechnology, Shanghai, China). The samples were separated electrophoretically on 10% SDS-PAGE gels for fractionation at 80 V for the first time and then at 120 V. After fractionation, the protein was transferred to a polyvinylidene difluoride (PVDF) membrane (Millipore, Bedford, MA, USA) at 200 mA for 2 h. These membranes were then blocked in 5% skimmed milk powder in TBS containing 0.1% Tween 20 (TBST) for 1 h at room temperature and then incubated at 4°C overnight with different primary antibodies. The primary antibodies were anti-p65 (^#^8242, 1 : 1000, Cell Signaling Technology (CST), Inc., Danvers, MA, USA), p-P65 (^#^3033, 1 : 1000, CST), p-I*κ*B*α* (^#^9246, 1 : 1000, CST), p-IKK*α*/*β* (^#^2697, 1 : 1000, CST), p-MEK (^#^9154, 1 : 1000, CST), MEK (ab178876, 1 : 20000, Abcam, United states), p-ERK (^#^3958, 1 : 1000, CST), ERK (^#^4695, 1 : 1000, CST), p-JNK (^#^9255, 1 : 1000, CST), JNK (^#^9252, 1 : 1000, CST), p38MAPK (AM065-1, 1 : 1000, Beyotime, Shanghai, China), p-AKT (^#^4060, 1 : 2000, CST), AKT (^#^4691, 1 : 1000, CST), VEGFA (ab1316, 1 : 100, Abcam), TGF-*β*_*1*_ (21898-1-AP, 1 : 600, Proteintech), and GAPDH (10494-1-AP, 1 : 10,000, Proteintech). After washing with TBST for 3 times, the membranes were incubated with horseradish peroxidase-conjugated secondary antibody (goat anti-rabbit IgG or goat anti-mouse IgG) for 1 h at room temperature. Finally, the immunoreactivity bands were detected by the enhanced chemiluminescence (ECL; Amersham Biosciences, Little Chalfont, UK) agent. The band intensities were analyzed using the Image J software. The protein expression of GAPDH was used as an internal control to normalize the data.

### 2.12. Immunohistochemistry Analysis of VEGFA Expression

Immunohistochemistry was performed to detect the protein expression level of VEGFA to determine the formation of new blood vessels at different days after injury. The operation process was as follows: the wound tissue section is kept at 60°C for 2 h and then deparaffinized and dehydrated. Thereafter, 3% H_2_O_2_ in methanol was used to prevent endogenous peroxidase activity. The section was added to anti-mouse VEGFA monoclonal antibody (ab1316, 1 : 200, Abcam) and incubated overnight at 4°C. After washing the sections with phosphate buffered saline, they were treated with horseradish peroxidase-conjugated secondary antibody (Wuhan Servicebio Technology Co., Ltd., China) at room temperature for 50 minutes. The section was examined under a microscope (100x magnification) to determine the positive expression of VEGFA in the wound. Then, the highest expression areas were observed under a microscope (400x magnification).

After being washed with phosphate buffered saline, the sections were treated with the secondary antibody conjugated with horseradish peroxidase (Wuhan Servicebio Technology Co., Ltd., China) at room temperature for 50 min. After staining with 3, 3′-diaminobenzidine (DAB)/H_2_O_2_ and hematoxylin, the sections were dehydrated, cleared, and mounted for viewing. For VEGFA analysis, the sections were examined under a microscope (100x magnification) to identify the highest positive expression in the wound. Then, the highest expression areas were observed under a microscope (400x magnification).

### 2.13. Statistical Analysis

The results were expressed as mean ± standard deviation (SD). The statistical significance of the detected differences was calculated by one-way analysis of variance (ANOVA) followed by Dunnett's multiple comparison test. Data analysis was performed by using SPSS 20.0 (SPSS Inc., Chicago, IL, USA) statistical package program. *P* < 0.05 was considered statistically significant, and *P* < 0.01 was considered highly significant.

## 3. Results

### 3.1. Effect of *D. dao* on Wound Closure and Percent Wound Contraction

**Figure 2:**
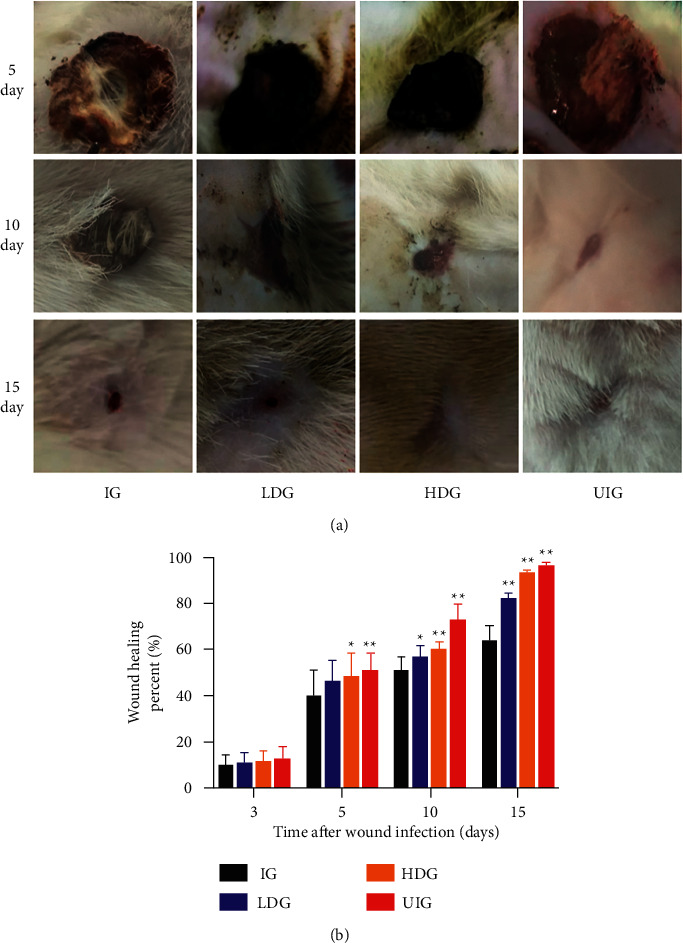
Effect of *D. dao* on gross appearance of healing wound and percentage of wound contraction (calculated in respect to day 0) on days 5, 10, and 15 after wounding in rats. (a) The other groups showed progressively better wound closure, as compared to the IG group. (b) Percentage of wound contraction in the other groups was greater, as compared to control. All values are represented as mean ± SD, *n* = 6 animals in each group. Significance was determined by ANOVA followed by Dunnett's test. ^*∗*^*P* < 0.05 and ^*∗∗*^*P* < 0.01 vs. IG group.

Early formation and shedding of scab, as well as wound closure, in *D. dao*-treated groups was characterized in comparison with IG (model) group. On the fifth day, local vasoconstriction created the local wound, most of which was filled with blood. The granular granulation tissue was not observed macroscopically. The normal wound group was moist and clean, with fresh particles around. On the 10th day, most of the wounds in the model group were covered by gray granulation tissue, the crust was hard, the depression was in the center of the wound, the recovery remained not ideal. The granulation tissue fully filled the bottom of the wound, and the epithelial tissue of the outer edge of the wound grew vigorously. On the 15th day, the wounds of the high-dose group of the traditional Chinese medicine group and the normal-wound group of the traditional Chinese medicine group were overall healed, and the rats exhibited a good condition. The wounds of the infection-wound group and the low-dose group of Chinese medicine were covered by scar tissue. Gross evaluation of wound suggested that topical *D. dao* application downregulated the wound size (Figure 2(a)) with significant percentage of increase in wound contraction (Figure 2(b)), as compared with IG group.

### 3.2. Effect of *D. dao* in Histopathology of Skin Tissue

**Figure 3:**
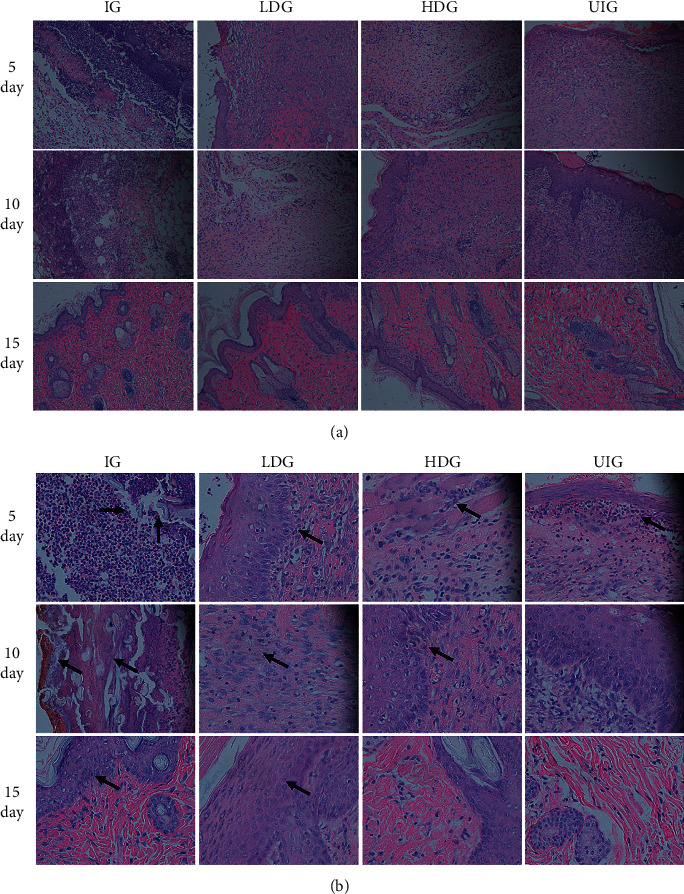
Representative images of H&E-stained histologic wound sections of IG, LDG, HDG, and UIG groups on days 5, 10, and 15 after wounding (x10 magnification shown in (a) and x40 magnification shown in (b)). H&E staining sections at 5, 10, and 15 days show that the other groups accelerated to the recovery of epidermis and reconstruction of skin appendage as compared to the IG group.

On the fifth day, considerable inflammatory factors infiltrated in each group, the tissue structure was necrotic, and the skin appendage disappeared. On the 10th day, the normal-wound group and the traditional Chinese medicine group showed the epidermis hyperplasia, the acanthosis was thickened, and the wound was attached to inflammatory cells. The number turned smaller, and the infective-wound group still showed significant inflammatory cell infiltration. On the 15th day, most of the epithelial tissues of the normal group and the traditional Chinese medicine group were well formed, and the epidermis was differentiated largely, close to the normal epithelium. The infective-wound group exhibited thicker epidermis than the normal epidermis, which was still accompanied by inflammatory cell infiltration. Low magnification (x10) images of wounds are shown in Figure 3(a) and the high magnification (x40) images are shown in Figure 3(b).

### 3.3. Effect of *D. dao* on Proinflammatory Mediators (pg/ml protein)

**Figure 4:**
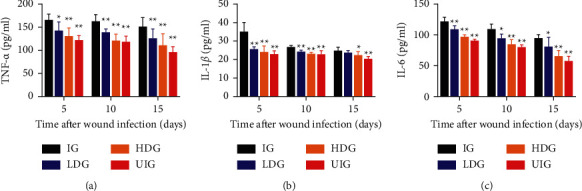
*D. dao* inhibits proinflammatory mediator expression and production in granulation/healing tissue of rats on days 5, 10, and 15 after wounding. (a) The expression of TNF-*α*. (b) The expression of IL-1*β*. (c) The expression of IL-6. Data were expressed as mean ± SD (*n* = 6). Significance was determined by ANOVA followed by Dunnett's test. ^*∗*^*P* < 0.05 and ^*∗∗*^*P* < 0.01 vs. IG group.

Serum levels of TNF-*α*, IL-1*β*, and IL-6 were measured in this study. As shown in Figure 4, the serum levels of TNF-*α* (Figure 4(a)), IL-1*β* (Figure 4(b)), and IL-6 (Figure 4(c)) were significantly increased in the IG group compared with the UIG group (*P* < 0.01).Conversely, compared with the IG group, the *D. dao* high-dose group and low-dose group could dramatically decrease the serum levels of TNF-*α*, IL-1*β*, and IL-6 (*P* < 0.05 or *P* < 0.01) on day 5, day 10, and day 15.

### 3.4. Effect of *D. dao* on Growth Factors

**Figure 5:**
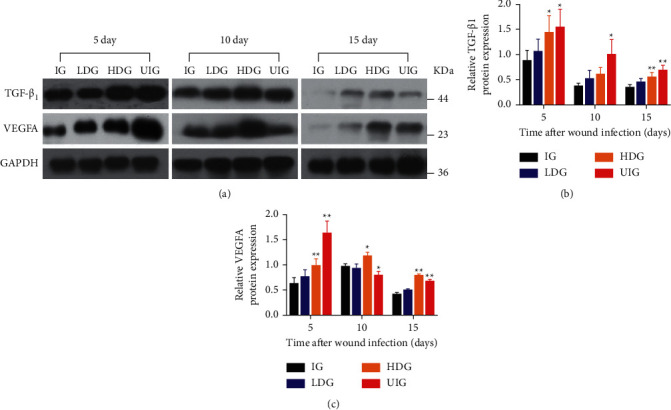
Effects of *D. dao* on the protein expression of TGF-*β*1 and VEGFA. (a) The western blot bands of TGF-*β*1 and VEGFA. (b) The relative protein expression of TGF-*β*1. (c) The relative protein expression of VEGFA. These proteins were normalized by GAPDH at each time point and values are expressed as relative change compared to IG group. Data were expressed as mean ± SD (*n* = 3). Significance was determined by ANOVA followed by Dunnett's test. ^*∗*^*P* < 0.05 and ^*∗∗*^*P* < 0.01 vs. the IG group.

The representative western blot bands of GAPDH, VEGFA, and TGF-*β*_1_ are given in Figure 5(a). In HDG group, the protein levels of VEGFA (Figure 5(b)) and TGF-*β*_1_ (Figure 5(c)) significantly increased on day 5 (1.00 ± 0.12-fold for VEGFA, 1.49 ± 0.33-fold for TGF-*β*_*1*_), day 10 (1.00 ± 0.12-fold for VEGFA, 0.62 ± 0.13-fold for TGF-*β*_*1*_), and day 15 (1.00 ± 0.12-fold for VEGFA, 0.56 ± 0.08-fold for TGF-*β*_*1*_); these levels increased in LDG group on day 5 (0.77 ± 0.13-fold for VEGFA, 1.07 ± 0.24-fold for TGF-*β*_*1*_), day 10 (0.94 ± 0.08-fold for VEGFA, 0.53 ± 0.16-fold for TGF-*β*_*1*_), and day 15 (0.51 ± 0.02-fold for VEGFA, 0.46 ± 0.06-fold for TGF-*β*_*1*_) after wounding in comparison with those of IG group.

**Figure 6:**
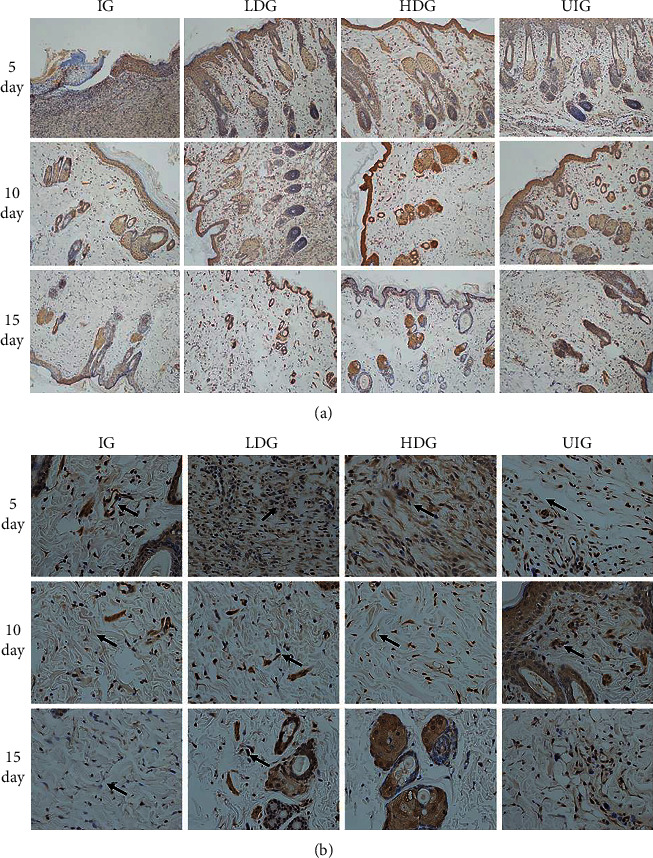
Representative images of immunohistochemistry analysis of the effect of *D. dao* on the expression of VEGFA in IG, LDG, HDG, and UIG groups on days 5, 10, and 15 after wounding (x10 magnification shown in (a) and x40 magnification shown in (b)).

Moreover, immunohistochemistry microscopy was performed to verify whether treatment with *D. dao* could upregulate VEGFA expression. Low-magnification (x10) images of wounds are shown in Figure 6(a) and the high-magnification (x40) images are shown in Figure 6(b). On day 5, VEGFA-related expression was observed everywhere in inflammatory cells, neutrophils, and lymphocytes, and in endothelial cells and fibroblasts; VEGFA expression in IG group was found significantly lower than that in other groups. On days 10 and 15, it was observed that VEGFA expression in endothelial cells and fibroblasts was significantly reduced.

### 3.5. Effect of *D. dao* on NF-*κ*B Signaling

**Figure 7:**
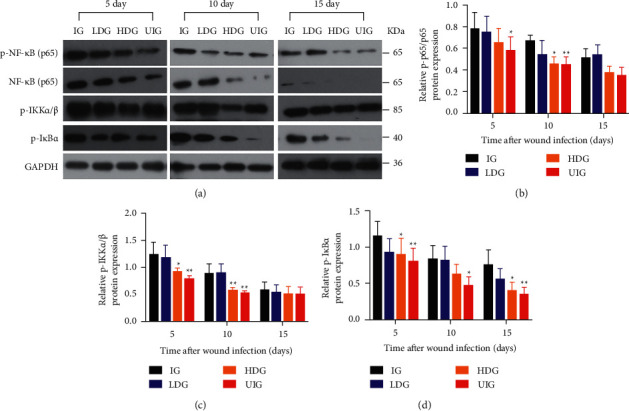
*D. dao* inhibits NF-*κ*B signaling pathway. (a) The western blot bands of p–NF–*κ*B (p65), NF-*κ*B (p65), p-IKK*α*/*β*, and p-I*κ*B*α*. (b) The relative protein expression of p-p65/*p*65. (c) The relative protein expression of p-IKK*α*/*β*. (d) The relative protein expression of p-I*κ*B*α*. Data were expressed as mean ± SD (*n* = 3). Significance was determined by ANOVA followed by Dunnett's test. ^*∗*^*P* < 0.05 and ^*∗∗*^*P* < 0.01 vs. IG group.

Whether *D. dao* affected NF-*κ*B signaling was examined. *D. dao* effectively inhibited degradation of the inhibitory proteins I*κ*B*α* and p-I*κ*B*α* in a dose-dependent manner (Figure 7(a)). Likewise, the phosphorylation of both p–NF–*κ*B p65 and NF-*κ*B p65 was inhibited by *D. dao* in a dose-dependent manner (Figure 7(b)). The phosphorylation status of IKK*α*/*β* proteins was also examined, namely, the upstream of the p-I*κ*B*α*/NF-*κ*B complex. IKK*α*/*β* protein phosphorylation was dramatically upregulated by infection stimulation, and this phosphorylation was significantly inhibited through rhododendrin treatment [28] (Figures 7(c)and 7(d)). These results imply that the NF-*κ*B signaling pathway is affected by *D. dao*, rendering it a useful anti-inflammatory therapy.

### 3.6. Effect of *D. dao* on MAPK and PI3K/Akt Signaling

**Figure 8:**
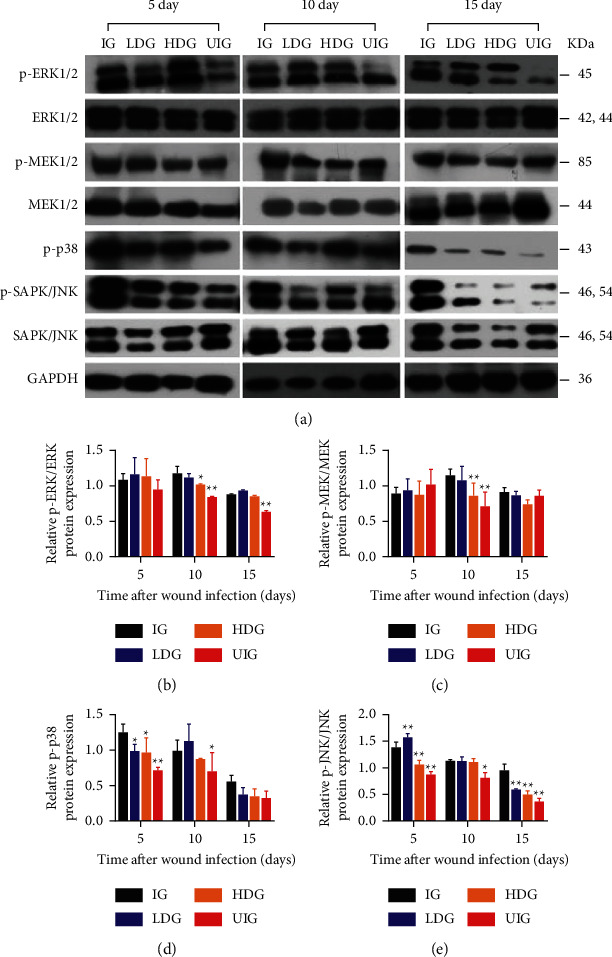
*D. dao* inhibits MAPK signaling pathway. (a) The western blot bands of p-ERK1/2, ERK1/2, p-MEK, MEK, p-p38, and JNK. (b) The relative protein expression of p-ERK/ERK. (c) The relative protein expression of p-MEK/MEK. (d) The relative protein expression of p-p38. (e) The relative protein expression of p-JNK/JNK. Data were expressed as mean ± SD (*n* = 3). Significance was determined by ANOVA followed by Dunnett's test. ^*∗*^*P* < 0.05 and ^*∗∗*^*P* < 0.01 vs. the IG group.

**Figure 9:**
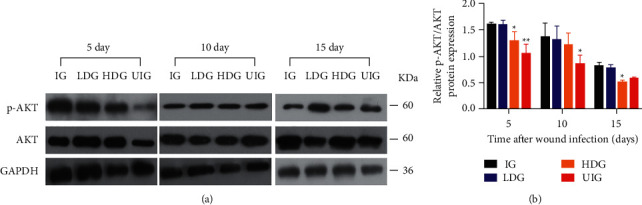
*D. dao* inhibits PI3K/Akt signaling pathway. (a) The western blot images of p-Akt and Akt. (b) The relative protein quantification in granulation/healing tissue of rats on days 5, 10, and 15 after wounding. These proteins were normalized by GAPDH at each time point and values are expressed as relative change compared to IG group. Data were expressed as mean ± SD (*n* = 3). Significance was determined by ANOVA followed by Dunnett's test. ^*∗*^*P* < 0.05 and ^*∗∗*^*P* < 0.01 vs. the IG group.

Whether *D. dao* could inhibit MAPK (Figures 8(a) and 8(b)) and PI3K/Akt (Figures 9(a) and 9(b)) signaling pathways, which were also activated by infection stimulation, was determined as well. In terms of MAPK signaling, *D. dao* effectively inhibited phosphorylation of ERK1/2, MEK, p38, JNK, and Akt in HDG group. These results imply that *D. dao* can inhibit MAPK and PI3K/Akt signaling, as well as NF-*κ*B signaling.

## 4. Discussion

This study aims to investigate the antimicrobial activity and anti-inflammatory effects of *D. dao* on the *E. coli*-treated wound skins under the simulated space environment. The results show that *D. dao* could significantly increase the healing rate of infectious wounds in rats, thereby shortening healing time, promoting the growth of granulation tissue, reducing inflammatory factor infiltration, promoting epidermal division, and exerting antibacterial effects. This study establishes an infectious wound model in rats on the simulation space environmental drug screening platform, which provides a reference for preparing a diseased animal model that is more fitted with the aerospace environment. The results of the experimental research revealed the antimicrobial activity and anti-inflammatory effects, as well as possible mechanism, of *D. dao* on bacterial infection wounds in rats under simulated space environment. This study provides an experimental basis for the development of *D. dao* as a bacterial infectious disease drug in the state of the simulated space environment.

Trauma may occur during long-term missions in the space station, such as tearing and open fractures. Wound healing is a process of simultaneous interaction of various cytokines and growth factors, disorder and change of which will result in damage to wound healing [29]. Normal wound healing consists of four stages: hemostasis, inflammation, proliferation, and remodeling. Bacterial infection is one of the main complex factors affecting wound healing by affecting several inflammatory and growth factors [30, 31].

*D. dao* is a traditional Chinese medicine with a long history for treating bedsore, ulcer, skin ulcer, and other infection diseases, main active ingredients of which are flavonoids and phenolic acids, which have significant antimicrobial, anti-inflammatory, and anti-infective activities [18]. In this study, we demonstrated that topical application of *D. dao* under simulated space environment can correct disordered infection wound healing by balancing the expression of various inflammatory mediators and growth factors during healing. Figure 2(b) shows that, compared with IG group, the wound healing rate of HDG group increased significantly from 5th day to 15th day. The results showed that the wound healing rate was significantly accelerated after *D. dao* application. On day 15, the wound almost completely healed. Compared with IG group, the expression of angiogenesis-related factors such as VEGFA and TGF-*β*1 was upregulated in HDG group, while the expression of inflammatory factors (TNF-*α*, IL-6, and IL-1*β*) was downregulated.

Granulation tissue is the basis for the formation of appropriate healing matrix, which is an immature type composed of inflammatory cells, angioblasts, fibroblasts, collagen fibers, and new blood vessels in the early stage [32, 33]. This immature granulation tissue becomes more mature and durable in the later stage, which is the basic feature of appropriate wound repair [34]. In addition to the formation of ECM during healing, its progressive degradation and remodeling must form mature wound healing tissues in a regulatory manner. The formation and programmed degradation of different cells and components in granulation tissue are influenced by different cytokines (such as TNF-*α*, IL-6, and IL-1*β*) and growth factors (such as TGF-*β*_*1*_ and VEGFA) [35]. Therefore, there should be a sufficient balance between synthesizers and degradants. Collagen is the main component of granulation tissue. The collagen synthesized by fibroblasts depends on TGF-*β*1, which is very important for wound healing [36]. It is reported that the application of TNF-*α* reduced the expression of collagen and the tensile strength of wounds [37]. Therefore, the increase of TNF-*α* level in later stage is harmful to granulation tissue. However, TGF-*β*_*1*_ is an important factor in the formation of high-quality granulation tissue. If the wound is filled with high-quality granulation tissue, the epithelium begins to form, because the granulation tissue provides a bed for laying the epithelium. Reepithelialization contributes to wound closure by transforming the keratinocytes from stationary phenotypes to migratory and proliferative phenotypes, which are impaired in bacterial infections. This may result from poor-quality granulation tissue and/or failure of keratinocytes transformation. The application of *D. dao* in infection wounds increased the expression of TGF-*β*_*1*_ (Figure 5(a)) and decreased the expression of TNF-*α* (Figure 4(b)), which were beneficial to increase collagenous fiber to form granulation tissue and accelerate epithelial regeneration during wound healing.

Angiogenesis is a part of the proliferative phase in wound healing, which involves the migration and proliferation of endothelial cells and angiogenesis. This process begins as early as the third day after injury [37]. VEGFA is a key regulator of many wound healing events, including angiogenesis, epithelialization, and collagen deposition [38]. In addition, it induces vasodilation, endothelial cell migration, and endothelial cell proliferation [39]. Here, we report that, after *D. dao* treatment, the expression of VEGFA in the wound was strongly induced on the 5th day due to bacterial proliferation. However, the expression of VEGFA ©n IG group was significantly lower than that in other groups. Compared with the ^5t^h day, inflammatory cells in LDG and HDG groups were mainly absorbed by wound tissue, and significant reductions in VEGFA expression were also observed in endothelial cells and fibroblasts on the 1^0t^h and 1^5t^h day (Figures 5(a) and 5(c)). The results of WB protein banding expression were consistent with those of immunohistochemistry.

Several cytokines (IL-1*β*, TNF-*α*, and IL-6) have been recognized as representative proinflammatory cytokines in wound healing. TNF-*α* in the inflammatory stage of wound healing can stimulate the chain reaction of inflammation by inducing and activating the expression and production of inflammatory cytokines such as IL-1 and IL-6 [40]. It participates in the initiation of early wound healing response. In wound healing, it can improve tissue repair by promoting the activation of inflammatory cells and regulating immune function as well as accelerating the migration and proliferation of keratinocytes. However, overexpression and imbalance of the relationship with other cytokines will cause a series of inflammatory damage. IL-1*β* is also a proinflammatory factor produced by macrophage activation, which can stimulate the effect of heat production and promote wound growth and healing and plays a key role in inflammatory response [40]. As the earliest and most important cytokine in inflammation, TNF-*α* can promote the formation of IL-1, and IL-1 can also induce the release of TNF-*α*. IL-6 is a multicellular cytokine with a wide range of biological activities, which participates in immune response, inflammatory response, and anti-infection defense. IL-6 promotes gene expression in the nucleus of inflammatory cells, as well as activation and aggregation of neutrophils, and induces the production of acute reactive protein in liver tissue through binding to receptors to activate the signal transduction pathway of MAPK, which is an important indicator reflecting the severity of inflammation and tissue injury, and its expression is positively correlated with the degree of trauma [41]. The level of IL-6 in patients with severe multiple trauma increased significantly, especially in patients with coinfection, so it can be used as one of the indicators to judge the degree of inflammation [31]. The results of this study showed that proinflammatory factors showed dynamic changes in the process of wound healing. Longitudinal comparison showed a downward trend at three time points, indicating that the inflammatory state was intrinsically related to wound healing. In the course of the disease, proinflammatory factors, such as TNF-*α*, IL-6, and IL-1*β*, showed synergy, which may be related to the abovementioned inflammatory chain reaction. The expression of TNF-*α*, IL-6, and IL-1*β* in IG group was significantly higher than that in normal group at each time points, which was negatively correlated with wound healing time, indicating that pathological inflammation caused by high expression of proinflammatory factors was one of the causes of delayed healing of infection wounds. The expression of TNF-*α*, IL-6, and IL-1*β* in the two groups intervened by *D. dao* decreased significantly than that in IG group, and the healing rate increased significantly at the same time point, suggesting that the regulation of proinflammatory factors could affect the wound healing rate. In addition, the wound treated by *D. dao* regenerated epithelial layer in a short time due to the lower level of inflammation and the increased fibroblasts (H&E staining, Figure 3(a)), eventually resulting in faster wound closure.

**Figure 10:**
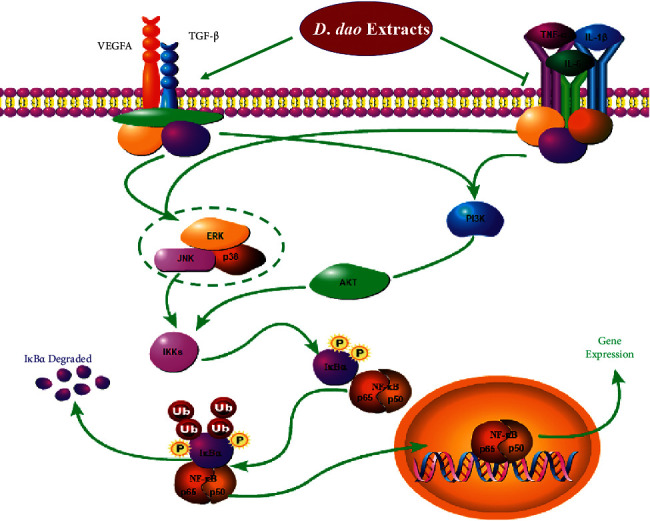
The summarized action pathways of *D*. *dao* on the infected wound.

To clarify the target pathway of *D. dao* therapy, we first studied the NF-*κ*B signaling pathway, namely, a major therapeutic target pathway activated by common inflammatory diseases. The binding of TNF-*α* to TNF receptor (TNFR) activates the NF-*κ*B pathway and induces chronic inflammatory diseases such as rheumatoid arthritis, inflammatory bowel disease, multiple sclerosis, and atherosclerosis [42, 43]. *D. dao* treatment reduced the degradation and phosphorylation of I*κ*B*α* and NF-*κ*B p65 and inhibited the phosphorylation of upstream signal protein IKK*α*/*β*, suggesting that *D. dao* affected the formation of IKK complex in the NF-*κ*B signaling pathway (Figure 7). Because inflammation is a complex network involved in multiple signal cascades, we also studied the effects of *D. dao* on other signaling pathways. It effectively inhibits signal transduction pathway of MAPK and PI3K/Akt (Figures 8 and 9). The signal pathways were summarized and presented in Figure 10. Our results suggest that *D. dao* is related to the inhibition of proinflammatory mediators, the enhancement of growth factor expression, and the inhibition of NF-kappa B, MAPK, and PI3K/Akt signaling pathways. In conclusion, these results support the potential use of *D. dao* as a novel local anti-inflammatory agent for wound infection (Figure S1: preliminary phytochemical study of *D. dao*. The mass spectrum of L-epicatechin (a), syringic acid (b), catechin hydrate (c), quercetin (d), gallic acid (e), methyl gallate (f), ethyl gallate (g), apigenin (h), and naringenin (i)).

## 5. Conclusion

The results reveal that *D. dao* application accelerates the timely progression of infective wound healing by regulating the expression of a number of cytokines and growth factors, including TNF-*α*, IL-6, IL-1*β*, VEGFA, and TGF-*β*_*1*_. It reduces inflammation and angiogenesis and improves maturation of infective wound. The mechanistic studies demonstrate that *D. dao* inhibits NF-*κ*B signaling as well as MAPK and PI3K/Akt signaling pathways. In conclusion, *D. dao* has shown potential in the treatment of cutaneous wounds in infective wound and it could be envisioned as a new agent for accelerating infective wound healing in the space station.

## Data Availability

The datasets used and/or analyzed in this study can be obtained from the corresponding author upon reasonable request.

## Abbreviations

*D. dao*: *Dracontomelon dao*
EtOAc: Ethyl acetate
*E. coli*: *Escherichia coli*
*P. aeruginosa*: *Pseudomonas aeruginosa*
*S. aureus*: *Staphylococcus aureus*
IL-6: Interleukin-6
IL-1*β*: Interleukin-1*β*
TNF-*α*: Tumor necrosis factor-*α*
VEGFA: Vascular endothelial growth factor A
TGF-*β*1: Transforming growth factor-*β*
NF-*κB*: Nuclear factor kappa-B
PI3K: Phosphoinositide 3-kinase
MAPK: Mitogen-activated protein kinase
IG: Infection group
LDG: Low-dosage group
HDG: High-dosage group
UIG: Uninfection group
HE: Hematoxylin-eosin
ELISA: Enzyme-linked immunosorbent assay
PMSF: Phenylmethylsulfonyl fluoride
BCA: Bicinchoninic acid assay
PVDF: Polyvinylidene difluoride
SD: Standard deviation
ANOVA: One-way analysis of variance.

## Supplementary Materials

Supplementary Materials Supplemental File 1: preliminary phytochemical study of *D. dao.*
