# Supplementary material for: Wound Healing Effects of Dracontomelon dao on Bacterial Infection Wounds in Rats and Its Potential Mechanisms under Simulated Space Environment
Source: Evid Based Complement Alternat Med. 2022 Jun 24;2022:4593201. doi: 10.1155/2022/4593201 (PMC9249481; doi:10.1155/2022/4593201)
Supplement: Supplementary Materials — Supplemental File 1: preliminary phytochemical study of D. dao. [file 4593201.f1.docx]

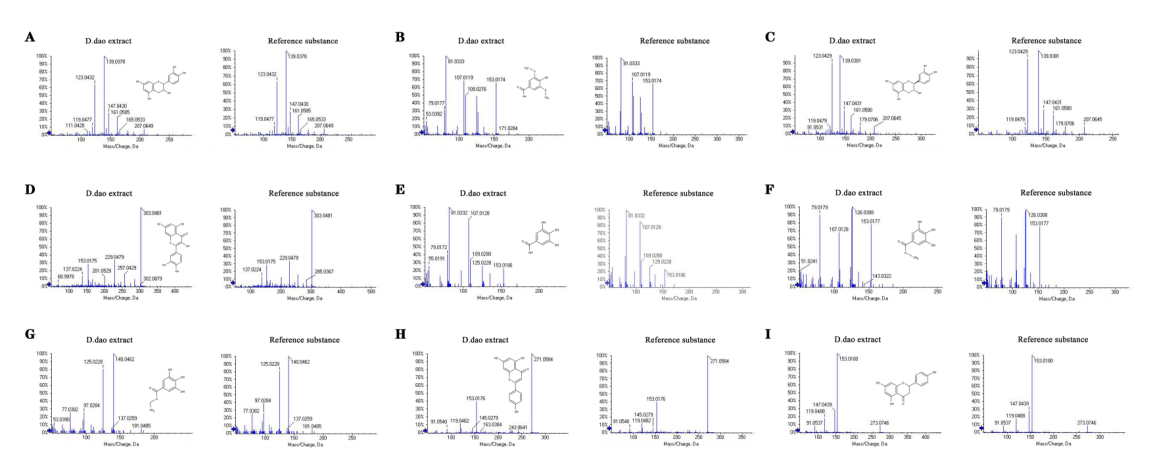


Fig. S1. Preliminary phytochemical study of *D.dao*. The mass spectrum of L-epicatechin (A), syringic acid (B), catechin hydrate (C), quercetin (D), gallic acid (E), methyl gallate (F), ethyl gallate (G), apigenin (H) and naringenin (I).
